# Measuring the impact of medical education in resource limited settings: A scoping review

**DOI:** 10.1371/journal.pgph.0004637

**Published:** 2025-08-05

**Authors:** Emily M. Jones, Eloise Stanton, Shervin Etemad, Alyssa Bautista, Jonathan Diaz, Elizabeth Cote, William P. Magee III, Allyn Auslander

**Affiliations:** 1 Department of Research, Operation Smile Inc, Virginia Beach, Virginia, United States of America; 2 Department of Plastic & Reconstructive Surgery, Keck School of Medicine, Los Angeles, California, United States of America; 3 Department of Global Programs, Operation Smile Inc, Virginia Beach, Virginia, United States of America; University of Global Health Equity, RWANDA

## Abstract

The Lancet Commission on Global Surgery identified workforce training as a key area of investment to improve access to essential surgical care. Many non-governmental organizations and universities have attempted to contribute to training and upskilling of providers in resource limited settings through educational programs. However, there are no widely agreed upon metrics for measuring the long-term success of these programs. A scoping review was conducted to assess varying methods used to measure impact, with a specific interest in patient-level impact. This scoping review was conducted in four databases (PubMed, Scopus, Embase, and Web of Science) following the PRISMA-ScR guidelines. The database search retrieved 1504 articles, of which 32 were included that quantitatively evaluated impact of medical education programs. Of the 32 articles, 6 measured patient-level impact defined by increased patient volumes after the education program or improved outcomes (decreased complications and mortality rates). The remaining 26 articles focused on provider-level impact primarily defined by skill acquisition and retention, as well as career advancement as a result of increased training. Provider-level impact was mostly assessed within 12 months of the program while patient-level impact was assessed longer after the program. There is a need to improve and standardize tools for measuring the impact of medical education. Patient impact should be the primary metric to evaluate the effectiveness of an educational program, and future tools should consider the long-term impact of training on the whole surgical workforce as opposed to a singular specialty.

## Introduction

Essential surgery is not accessible to most of the world’s population, and often causes impoverishing or catastrophic expenditure for those who are able to access it [1,2]. In 2015, the Lancet Commission on Global Surgery (LCoGS) identified workforce training and development as a key factor to improve access to surgical care, recommending 20 general surgeons, anesthesiologists, and obstetricians (SAO) per 100,000 population to meet this need [2]. Currently, the SAO density in low- and middle-income countries (LMICs) is 3.19 per 100,000 population, which falls substantially short of the LCoGS benchmark [1,2]. In addition to SAOs, LCoGS recommends including nurses, community health workers, biomedical technicians, and more, in workforce development to encompass the entire surgical system [2]. To close this gap, non-governmental organizations (NGOs) and university partnerships are an effective way to provide educational opportunities for healthcare professionals that aim to upskill providers while providing direct patient care [3,4]. Methods to measure the effectiveness of these programs vary widely in the literature. Some studies use entrusted professional activity scales, such as the Accreditation Council for Graduate Medical Education core competencies, to assess the use of the training in the participants’ practices, but these types of assessments are not usually applicable for health professionals in LMICs that can range in experience from residents to fully-trained professionals being upskilled with a specific technique [5,6]. Other studies use procedure-specific competency scores immediately after and/or as a follow-up assessment to assess the program’s effectiveness in teaching a certain skill after the program [7,8]. Resource limitations and prioritization of service delivery over research activities, however, may limit the publication of programmatic activities by NGOs.

While skill retention, confidence, and career progression are useful metrics, they focus on provider outcomes which don’t necessarily quantify impact on patients. Other studies use morbidity, complication rates, and patient volumes to quantify the impact of education on patient care [9,10]. These metrics are difficult to clearly associate with a certain intervention which can cause an overstatement of impact [11]. With increased interest in improving access to medical education through NGOs and university partnerships, there is a growing need for standardized tools to define and measure the impact of these programs.

“Impact” has become a popular buzzword used for programmatic assessments or demonstration of program outcomes, but many impact assessments fall short in proving tangible values beyond confidence or skill retention [12]. This scoping review aims to assess what tools have been used to measure how education provided to health professionals by NGOs or university partnerships impacts patient care. Specifically, we seek to understand how educational interventions are evaluated as beneficial to patients or to the health facility that participated in the NGO- or partnership-provided training. Benefit here is defined as reduced morbidity, reduced complication rates, improved patient reported outcomes, and receiving care that previously didn’t exist. By synthesizing the available literature, this review highlights areas for improvement and suggests directions for future research to enhance the evaluation of healthcare education in LMICs and assess the efficacy of programs provided by outside groups.

## Materials and methods

**Fig 1:**
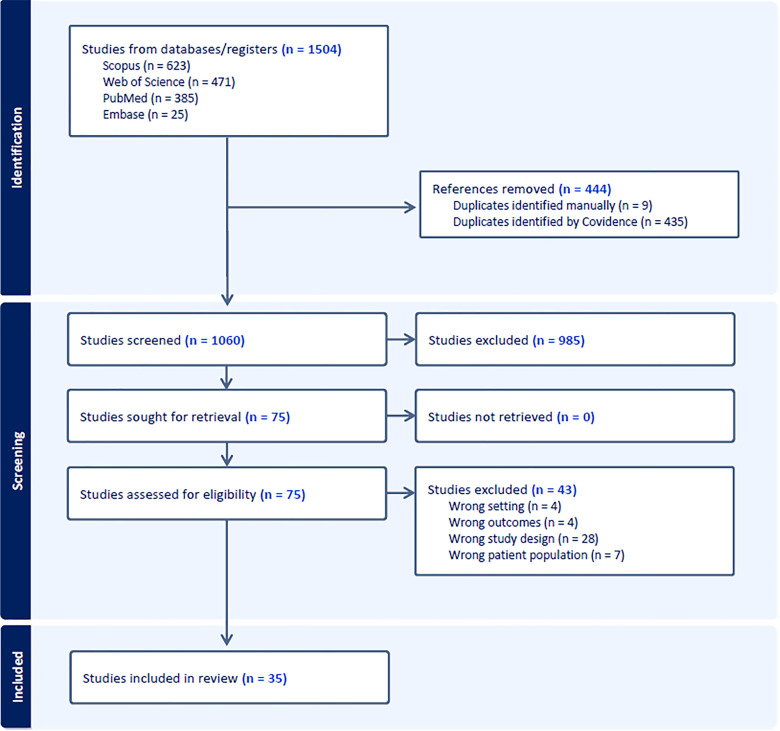
Preferred Reporting Items for Systematic Reviews and Meta-Analyses flowchart.

This scoping review was conducted following the Preferred Reporting Items for Systematic Reviews and Meta Analyses extension for Scoping Reviews (PRISMA-ScR) guidelines for reporting (S1 Checklist, Fig 1).

### Search strategy, study selection, and data collection

A literature search was conducted on May 8, 2024, across 4 databases: PubMed (National Institutes of Health, Bethesda, MD); Scopus (Elsevier, Amsterdam, the Netherlands); Embase (Elsevier); and Web of Science (Clarivate, London, UK). The search framework is shown in Table 1. Literature was in English language due to the authors’ limited language ability. No timeframe restrictions were applied. Inclusion criteria included original, peer-reviewed, full-text articles that reported quantifiable impact of NGO or university partnership training initiatives for surgeons, anesthetists, pediatricians, and nurses. Excluded were abstracts, conference proceedings, editorials, and studies that did not report quantified, numerical impact. Author ES conducted study identification, and all search results were screened by authors EJ and ES for adherence to inclusion criteria. Differences between authors were resolved through discussion. Full texts for included studies were read in full and assessed for content relevance by author EJ.

**Table 1:** Search strategy defined by population, concept, context framework, with Boolean operators.

**Population terminology**	Healthcare professionals, medical professionals, doctors, surgeons, nurses, healthcare workers
**Concept terminology**	Impact, evaluation
**Context terminology**	Education, training, NGO, Charity, mission, surgical program, surgical camp, non-for-profit
**Search strategy**	(“Education” OR “Training”) AND (“Impact” OR “Evaluation”) AND (“Healthcare professionals” OR “Medical professionals” OR “doctors” OR “surgeons” OR “nurses” OR “healthcare workers”) AND (“NGO” OR “Charity” OR “Mission” OR “Surgical program” OR “Surgical camp” OR “non-for-profit”)

### Synthesis of results

Study data was analyzed across four categories: involvement of NGO/university, training status of population studied, type of impact collected, and type of measured impact. Studies that reported impact through number of patients receiving care, morbidity and mortality rates, or complication rates were considered “patient impact.” Studies that reported impact through evaluations of training participants such as skill retention, confidence, motivation, influence on career, and skill sharing, were considered “provider impact.” Summative and descriptive statistics were used to describe the analysis of the studies included.

## Results

The initial search yielded 1504 results. After removing duplicates and applying inclusion/exclusion criteria, 75 studies underwent full text review. The search results and exclusions are shown in Fig 1. An additional 43 studies were excluded because of study characteristics, and 32 studies remained for data extraction and full text analysis.

Table 2 shows the demographics of included study participants: from LMICs (44%) and HICs (47%), and a mix of LMICs and HICs (9%). Studies occurred across a variety of education delivery settings including NGOs (44%), university partnerships (19%), and other settings (38%) which are mostly comprised of training programs adapted from organizations and conducted in a hospital. Study participants were diverse and included in-training medical professionals, independent medical professionals, and a series of other health care support workers who did not participate in direct care delivery.

**Table 2:** Study demographics of all included studies.

Study demographics, *n (%)*	Patient Impact (n = 6)	Provider Impact (n = 26)	*Total (N = 32)*
Study Setting			
LMIC	3 (50%)	11 (42%)	14 (44%)
HIC^+^	3 (50%)	12 (46%)	15 (47%)
Both	0 (0%)	3 (12%)	3 (9%)
Education context			
NGOs	2 (33%)	12 (46%)	14 (44%)
University partners^*^	2 (33%)	4 (15%)	6 (19%)
Other^^^	2 (33%)	10 (38%)	12 (38%)
Study participants			
In-training medical professionals	2 (33%)	10 (38%)	12 (38%)
Fully independent medical professionals	1 (17%)	10 (38%)	11 (34%)
Both in-training and fully independent medical professionals	0 (0%)	4 (15%)	4 (13%)
Non-medical professionals	0 (0%)	1 (4%)	1 (3%)
Patients or patient health records	3 (50%)	0 (0%)	3 (9%)
Unspecified	0 (0%)	1 (4%)	1 (3%)

+UMIC grouped with HIC.

*Partnership between HIC university and LMIC/LIC university or hospital.

^Programs adapted from NGOs or universities but not necessarily funded or hosted by NGOs or universities.

### Impact assessment

Impact was evaluated using pre-/post-intervention surveys (n = 4, 13%), post-intervention surveys alone (n = 25, 78%), and patient chart data (n = 3, 9%). Studies were further categorized based on whether the impact measured was directly related to patients or the medical providers (Table 2).

### Patient-level impact

Half of the studies that measured patient impact used patient health record data (n = 3, 50%) and were conducted in LMICs (n = 3, 50%). Studies that quantified patient impact primarily used surgical volume from before and after the educational intervention to determine if there was an increase in surgery (Table 3). Of the six studies that measured patient impact, four reported increased patient volume after the training programs. Table 4 shows the details of the patient impact papers included. The two papers reporting on plastic surgery reported 906 additional surgeries in 5–8 years at three hospitals and 474 additional surgeries in 15 years at one hospital [13,14]. One paper reporting on ophthalmology surgery reported 3 additional surgeries per trainee in one year after a simulation course [15]. One paper reporting on neurosurgery reported an average of an additional 43 surgeries between two surgeons in 8 years at one hospital [16].

**Table 3:** Studies measuring patient-level impact (N = 6, 19% of included studies).

		*n (%)*
**Study period post-intervention**	0-12 months	1 (17%)
	1-5 years	2 (33%)
	5-10 years	2 (33%)
	10 + years	1 (17%)
**Impact measurement** ^*^	Morbidity/Mortality rate	1 (11%)
	Complication rate	3 (33%)
	Patient volume	5 (56%)

*Some studies included multiple impact metrics which makes the values in this column add up to greater than the study inclusion size of 6.

**Table 4:** Details of included studies that measured patient impact.

Title	Author (Year)	Country	Number of participants	Timeframe (after program)	Impact calculated
Expansion of reconstructive surgical capacity in Vietnam: experience from the resurge global training program	Luan (2022)	Vietnam	2,018 cases	5 years	906 additional patients receiving surgery over 4 years
Simulation-based surgical education for glaucoma versus conventional training alone: the Glaucoma Simulated Surgery (GLASS) trial. A multicentre, multicountry, randomised controlled, investigator-masked educational intervention efficacy trial in Kenya, South Africa, Tanzania, Uganda and Zimbabwe	Dean (2021)	Kenya, South Africa, Tanzania, Uganda, and Zimbabwe	49 surgeons	3 months, 1 year, and 15 months	Intervention group did 3.2 live surgeries compared to non-intervention group that did 0.15 live surgeries.
Optimizing international neurosurgical outreach missions: 15-year appraisal of operative skill transfer in Lima, Peru	Jandial (2021)	Peru	2 surgeons	8-16 years	42.5 avg. cases during study period, compared to 3 cases before intervention
Implementation of a Progressive Mobilization Program in a Medical-Surgical Intensive Care Unit	Messer (2015)	USA	41 nurses	3 weeks	60% receiving mobility from 39% before intervention
Avoidable iatrogenic complications of male urethral catheterisation and inadequate intern training: A 4-year follow-up post implementation of an intern training programme	Sullivan (2014)	Ireland	725 patients	4 years	Decrease in complications: 6% to 4%. Decrease in morbidity when intern performing procedure: 74% to 62%
Introduction of microsurgery in Vietnam by a charitable organization: A 15-year experience	Merrell (2005)	Vietnam	474 patients	15 years	0 to 474 cases (over 14 years)

### Provider-level impact

26 of the included studies measured impact through provider impact defined as skill retention, confidence, career impact, or knowledge sharing. The majority of studies (81%) utilized a post-intervention survey only, while the rest utilized pre- and post-intervention surveys or post-intervention interviews. Most of the studies evaluated impact within 12 months of the educational intervention (69%) although a few studies evaluated impact after more than 10 years (7%). The most common survey tool was skills tests, particularly if the study involved in-training medical professionals. Studies that included self-reported confidence were used on both in-training and fully independent medical professionals Table 5.

**Table 5:** Studies measuring provider-level impact (N = 26, 81% of included studies).

		*n (%)*
**Study period post-intervention**	0-12 months	20 (69%)
	1-5 years	6 (21%)
	5-10 years	1 (3%)
	10 + years	2 (7%)
**Impact assessment** ^*^	Skill retention	15 (47%)
	Confidence	7 (22%)
	Career impact	3 (9%)
	Knowledge sharing	7 (22%)

*Some studies included multiple impact metrics which makes the values in this column add up to greater than the study inclusion size of 26.

## Discussion

Of the 1060 screened studies that included the word “impact,” only 32 included supporting evidence documenting quantitative impact measurement. While impact can also be demonstrated descriptively, the lack of supporting evidence for these statements is a major limitation of stated “impact.” Additionally, only 6 of the 32 included studies address patient-level impact, which represents 0.6% of all screened studies. The purpose of medical education is the ultimate translation to safe and accessible care, and we believe that the lack of evidence for statements of impact represents a major limitation of the literature on this topic.

Patient level impact is most clearly shown through change in patient volume before and after an educational program. The LCoGS identifies patient volume as a key metric to evaluate global surgical programs’ effectiveness in increasing access to care, however the authors acknowledge that an increase in surgical volume is restricted by the availability of necessary resources which are often restricted in LMICs which makes this variable confounded [2]. Four of the five volume-focused studies used change in surgical patient volumes as a result of training surgeons in a new skill or technique. The existence of surgeons to be trained assumes that there is also an established surgical workforce, including trained anesthesiologists, nurses, biomedical technicians, etc. as well as specialized instruments and equipment. When the new skills being taught to the surgeons do not necessitate additional training for supporting specialties, then a change in patient volume is a reasonably accurate metric to use. The only volume-focused study that did not involve surgical training focused on patients that received mobility care after a program with intensive care unit nurses compared to those who did not receive mobility care before the program [17]. Since the mobility exercises from the training program only require the patient and nurse, and no additional resources, this is the closest one-to-one impact assessment from all of the patient-level impact studies. However, this paper does not explore how mobility benefited the patients, such as improved wound healing or shorter hospital stays, which limits the definition of “impact” to whether care was given, and not the long-term outcomes of receiving this care. Although this research would be more complicated and require case-controls, it would be the next step to understanding the true impact of the educational program.

Patient-level impact assessment should go beyond the short-term outcomes to understand how an intervention creates change or affects a certain population [18]. Patient-level impact can also be assessed through complication and revision rates which can show the long-term outcomes of the training and whether a patient benefitted from receiving the care. One study included reconstructive flap failure rate as a reported outcome in addition to change in surgical volume over the span of 10 years after an educational intervention [14]. This is a more comprehensive and rigorous assessment of assessing both increase in access to care through patient volume as well as quality of care through postoperative outcomes.

Provider-level impact can also serve as a useful proxy for understanding the effectiveness of various initiatives. Pre-and post-tests can measure knowledge or skills gained through education [19]. Only four of the 26 studies on provider impact included a pre-test. All the studies showed skill acquisition after the education program, but the studies with pre-tests show *improved* skills which gives important context to the impact of the program: if the skills acquired are new, then the impact of the program could be greater than if those skills were just being reinforced. Additionally, skill retention is an important metric since surgical skills have been shown to decrease significantly if not retrained within one year [20,21]. Four of the included studies look at data at the conclusion of the program with additional assessment at 6 or 12 months after the program. Most of the provider-level studies assessed skills anywhere from a few days to 7 years after the education program, but there was not a consistent timeline for testing or retesting skills across the included studies. Although skills tests don’t necessarily indicate patient impact, they are still a useful measurement for understanding knowledge dissemination and could be more effective in demonstrating impact if consistent measurement tools and timelines existed.

Across all the studies, there is a lack of research that measures impact across surgical teams. Almost half of the studies focus solely on surgeons while only seven address multiple specialties. Although training tends to be specific to a certain specialty, the acquired skills or treatment are rarely performed in isolation of other support staff. For example, a surgeon performing a new surgery might require nurses to learn new post-operation procedures. Additionally, no studies quantified indirect impact as a result of the training participants passing their knowledge on to colleagues who can then use it in their practices. The generational impact of “training the trainer” captures the long-term or indirect patient impact of educational programs [11]. Improved impact assessments would include multiple specialties and would consider indirect patient impact.

One limitation of this study is that there is currently no universally agreed upon definition or measurement for what constitutes impact. Limiting a literature search to studies that explicitly use the word “impact” allowed for a more straightforward search criteria while being able to comment on the lack of agreement on what constitutes “impact”, but we recognize that there may be studies that address various levels of patient- and provider-level impact without the explicit use of the term. In addition, when considering the landscape of NGO-based healthcare training initiatives, published studies in peer-reviewed journals may miss data that is reported in other forums such as NGO annual reports, press releases, and other grey literature sources such as white papers and policy briefs, including reports and literature in languages other than English, which would have made this scoping review more inclusive.

## Conclusions

This scoping review highlights the limited availability of standardized tools for measuring the impact of education in NGO, university and hospital settings, particularly for healthcare professionals in low-resource environments. While many studies assessed provider impact, few measured direct patient outcomes. Those that do use patient outcomes may overestimate the contribution of the educational initiative to the stated outcome. Most studies used post-intervention surveys without long-term follow-up, leading to variability in the reported impact. These findings underscore the need for standardized, patient-centered metrics to better quantify the effectiveness of educational programs in these settings. Future research should focus on creating and validating consistent measurement tools to assess both immediate and long-term outcomes of healthcare training programs.

## Supporting information

S1 Checklist Preferred Reporting Items for Systematic reviews and Meta-Analyses extension for Scoping Reviews (PRISMA-ScR) checklist. (DOCX)
